# Competitive reduction of poultry-borne enteric bacterial pathogens in chicken gut with bioactive *Lactobacillus casei*

**DOI:** 10.1038/s41598-020-73316-5

**Published:** 2020-10-01

**Authors:** Zajeba Tabashsum, Mengfei Peng, Zabdiel Alvarado-Martinez, Arpita Aditya, Jacob Bhatti, Paulina Bravo Romo, Alana Young, Debabrata Biswas

**Affiliations:** 1 Biological Sciences Program - Molecular and Cellular Biology, University of Maryland, College Park, MD 20742 USA; 2 Department of Animal and Avian Sciences, University of Maryland, College Park, MD 20742 USA; 3 Center for Food Safety and Security Systems, University of Maryland, College Park, MD 20742 USA

**Keywords:** Microbiology, Antimicrobials, Applied microbiology, Pathogens

## Abstract

In this study, the effect of sustainable probiotics on *Campylobacter jejuni* colonization and gut microbiome composition was evaluated using chicken as a model organism. Chickens were given *Lactobacillus casei* over-expressing myosin-cross-reactive antigen (LC^+*mcra*^). LC^+*mcra*^ can generate bioactive compounds in larger quantity including conjugated linoleic acid. A total of 120 chickens were used in duplicate trials to investigate the effectiveness of LC^+*mcra*^ in decreasing *C. jejuni* colonization by means of kanamycin resistant strain compared to the control group. We observed that LC^+*mcra*^ can efficiently colonize various parts of the chicken gut and competitively reduce colonization of natural and challenged *C. jejuni* and natural *Salmonella enterica*. LC^+*mcra*^ was found to reduce *C. jejuni* colonization in cecum, ileum and jejunum, by more than one log CFU/g when compared to the no-probiotic control group. Furthermore, 16S rRNA compositional analysis revealed lower abundance of Proteobacteria, higher abundance of Firmicutes, along with enriched bacterial genus diversity in gut of LC^+*mcra*^ fed chicken. Decreased contamination of drinking water by *C. jejuni* and *S. enterica* was also observed, suggesting a potential function of reducing horizontal transfer of enteric bacteria in poultry. Outcomes of this study reveal high potential of LC^+*mcra*^ as sustainable approach to decrease colonization of *C. jejuni* and *S. enterica* in poultry gut along with other beneficial attributes.

## Introduction

*Campylobacter jejuni* causes foodborne enteric infection campylobacteriosis and is considered as one of the prominent zoonotic pathogens. During 2018, among over twenty-five thousand cases of foodborne infections reported by FoodNet, the incidence rate of *Campylobacter* infections was the highest, 19.5 in 1000 people^1^. Poultry is the major reservoir of *C. jejuni* and cross-contamination of chicken meat during processing causes the majority of sporadic occurrences of campylobacteriosis^1, 2^. The current trend of white meat consumption, particularly chicken meat, has been increased significantly in the US and around the world, which is believed to be the reason for the increasing numbers of campylobcateriosis^3^. *C. jejuni* is normally present in the gut microbiota of poultry and other birds, capable of growing optimally at 42 °C, which is the body temperature of chicken^4^. According to recent reports, the prevalence pattern of *C. jejuni* in pasture poultry and their sensitivity to various antibiotics indicate that animal farming without antibiotic and/or synthetic chemical growth promoters diminishes the prevalence of multiple antibiotic resistance *C. jejuni* in poultry, without altering colonization of other bacteria^5,6^. Another important zoonotic pathogen is *Salmonella enterica*, with a higher prevalence in organic farming systems than in conventional farming systems, but a lower incidence of multi-antibiotic resistance in their organic counterparts^7, 8^. Therefore, developing sustainable alternatives that can replace antibiotic growth promoters and synthetic chemicals is a priority in order to reduce multiple-drug resistant *C. jejuni* and *S. enterica*.

Probiotics can be considered as the prime candidates for the prevention and reduction of cross-contamination with foodborne bacterial pathogens in food products^8–12^, however, their effectiveness is heavily dependent on the variety and total amount of produced and secreted metabolites/byproducts^11,13^. Probiotics can break down undigested dietary components through colonizing in the gastrointestinal (GI) tract of the host and after reaching these undigested components in different portions of intestine, especially cecum, jejunum and ileum, the probiotics produce a remarkable quantity of metabolites or bacterial byproducts^14,15^. Probiotics and their metabolites also modulate the composition of gut microbiome by competitive colonizing or through the secretion of bioactive compounds in different gut intestinal portions^16,17^. For example, the increase of the beneficial microorganisms in gut microbial ecosystem ultimately decreases the detrimental microbiota, which can lead to a healthy gut state for the overall health of the host^16,17^.

The amount and types of bioactive metabolites produced by beneficial microbes are vastly influenced by undigested dietary components and/or prebiotics^11,13^. Any potential prebiotic intended for use has to comply with some regulations, like possessing the “Generally Recognized as Safe (GRAS)” status, being assessed for appropriate dose and potential side effects, must be free of contaminants and impurities and must not cause alterations to the intestinal microbiota that could negatively affect the host^18^. According to Wang, it is reported that prebiotics must not be digested in the upper portion of the gastrointestinal tract before reaching large intestine, where they are selectively fermented by beneficial and/or commensal intestinal microbes^19^. Such type of fermentation leads to the changes in metabolic processes of gut microbes and nutrients availability in gut ecosystem, thus possessing a beneficial effect on the host. But these beneficial effects vastly depend on the selective stimulation of intestinal probiotics with prebiotic^19^. With such regulations and conditions, it is difficult to find an ideal prebiotic. Instead of adding stimuli or prebiotic, designing a probiotic that can provide enhanced beneficial effects by itself is a better alternative approach^20^. Bacterial strains usually used for synbiotic include but not limited to *Lactobacillus* spp.,* Bifidobacteria* spp. and several prebiotics mostly form natural food sources like cocoa, peanut^21^.

The secondary metabolites produced by beneficial microbes include lipids, particularly short chain and poly-unsaturated fatty acids^22–25^. Among the short chain and poly-unsaturated fatty acids, linoleic acid (LA) is considered as one of the most functional metabolites produced by different probiotic species of *Bifidobacterium*, *Lactobacillus* and *Lactococcus*^26^. Several research groups, including our own, have focused on enhancing the productivity of LA and conjugated LA (CLA) from probiotic sources, both in the human/animal gut environment and the industrial production^11,13,27^. Our previous study showed a relative high number of *Lactobacillus* and their metabolites exhibited antimicrobial activities against various enteric pathogens such as *C. jejuni*, *S. enterica* and enterohemorrhagic *Escherichia coli*^28–31^. *Lactobacillus* spp. also have the adherence and multiplying ability in the host^32^. They can produce acids, hydrogen peroxides and bacteriocins, but with no pathogenic traits and can persist as normal flora^32^. Among different species of *Lactobacillus*, *L. casei* has been reported as one of the crucial probiotic strain^32^. Considering this, our research team over-expressed the myosin-cross-reactive antigen (Mcra) (‘*mcra*’ gene obtained from *L. rhamnosus*, another probiotic strain)^33^ for linoleate isomerase over-production in *Lactobacillus casei* (LC). The *mcra* over-expressing probiotic, named as “LC^+*mcra*^”, showed enhanced activities, including anti-inflammatory and antimicrobial effects both in vitro and in vivo animal model (mice)^13,31^. It has also been reported that LC^+*mcra*^ induced no detrimental effect on mice gut, rather it improved the murine gut health through the modulation gut microbiome^13^.

Here, we intended to evaluate the effectiveness of LC^+*mcra*^ on the colonization of a marker-containing *C. jejuni* strain (CJRMKm) as well as the natural colonization of *C. jejuni* and *S. enterica* in different sections of chicken gut. We also aimed to compare the gut microbial composition of the control and LC^+*mcra*^ fed group chickens through 16S rRNA compositional analysis. This study may help to evaluate the effect of LC^+*mcra*^ in controlling the colonization of *C. jejuni* and *S. enterica* in chickens at the pre-harvest level, along with the effect of LC^+*mcra*^ on modulation of gut microbiome.

## Results

### Efficient colonization capability of LC^+*mcra*^ in chicken gut

**Figure 1:**
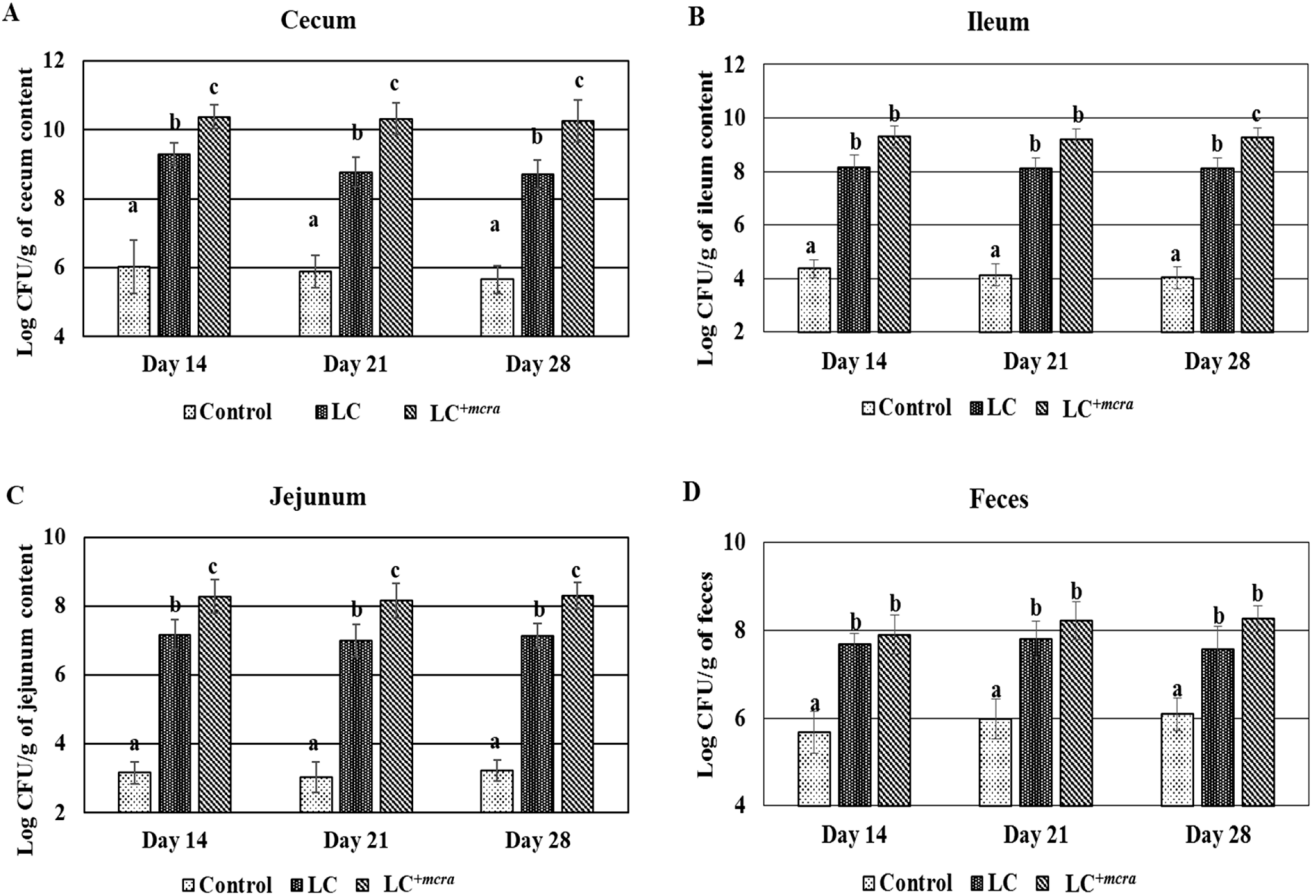
Colonization of wild-type probiotic, LC and CLA over-producing probiotic, LC^+*mcra*^ in different intestinal portions of chickens and their fecal shedding pattern compared to control in terms of *Lactobacillus* count. Colonization of LC/LC^+*mcra*^ in cecum (**A**), ileum (**B**), jejunum (**C**) and fecal shedding of LC/ LC^+*mcra*^ (**D**) in terms of *Lactobacillus* count. Results are mean ± SD and letters (a–c) are used to mark the significant differences at various time points compared with control and among the treatment groups at *p* < 0.05.

To compare the colonization capability of LC and LC^+*mcra*^ in various sections of chicken gut, the colonization of *Lactobacillus* was enumerated and compared in our three groups of chicks from two separate trials (Fig. 1). The administration with LC^+*mcra*^ showed higher and more stable cecal colonization level of *Lactobacillus,* above 10 log CFU/g throughout 4 weeks, which were substantially higher than wild-type LC fed group as well as natural colonization group (Fig. 1A). A similar pattern was found in LC^+*mcra*^ fed chicken jejunum, which showed a significantly (*p* < 0.05) higher number of *Lactobacillus* at day 28 (Fig. 1B). Though this difference was only numerically higher in ileum when compared to wild type LC fed group (Fig. 1C). The number of *Lactobacillus* in the fecal shedding of probiotic-fed group were observed to rise when compared to the control group throughout the study period (Fig. 1D). Specifically, in the chicken group fed with LC^+*mcra*^, colonies of *Lactobacillus* in fecal shedding gradually increased, whereas fecal shedding of *Lactobacillus* in the wild-type LC fed group and control group (group fed with no probiotic) were observed to be noticeably lower than that in the LC^+*mcra*^ fed group (Fig. 1D).

### Control of colonization of *C. jejuni* strain in probiotic fed chickens

**Figure 2:**
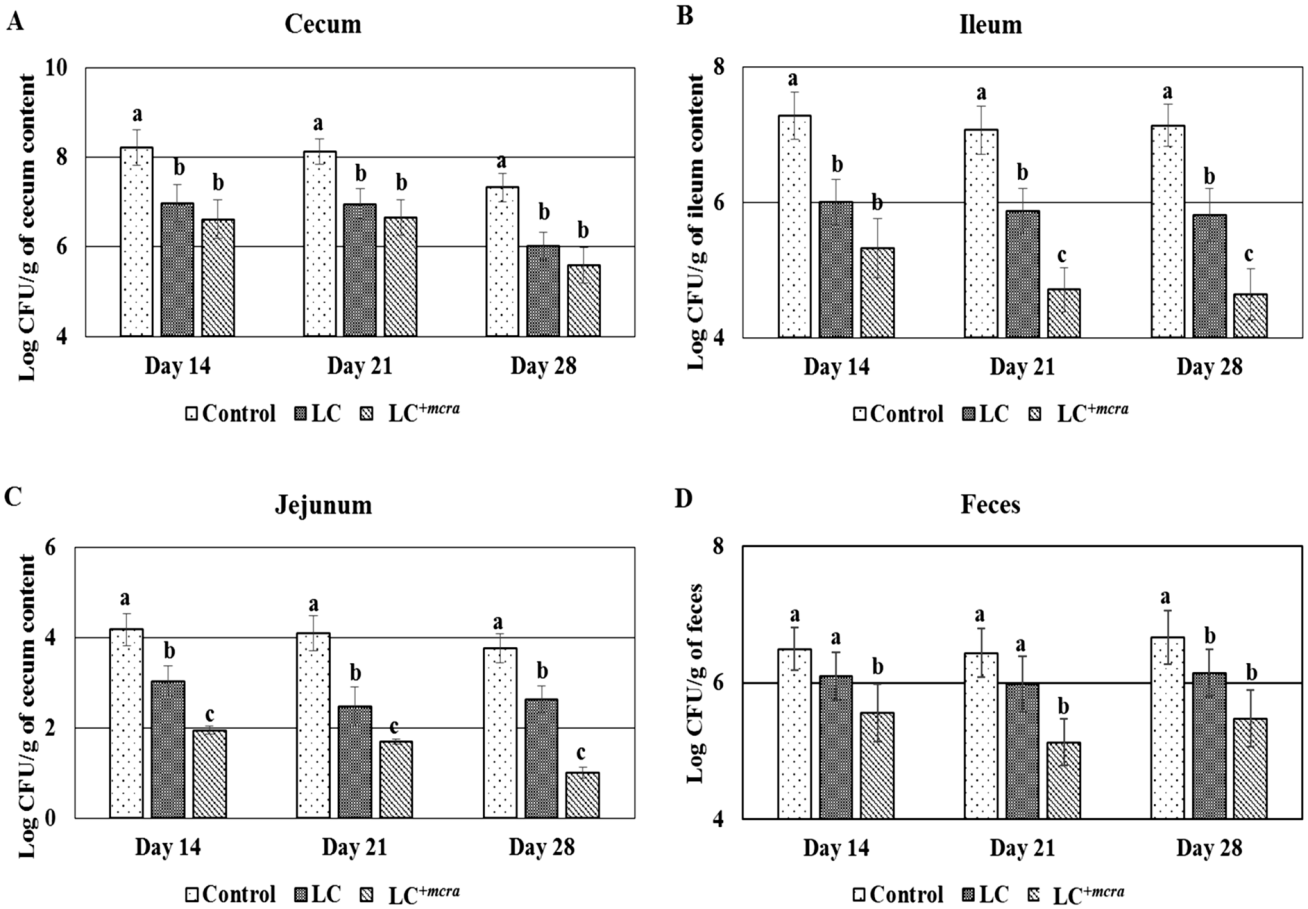
Effect of wild-type probiotic, LC and CLA over-producing probiotic, LC^+*mcra*^ on colonization and fecal shedding pattern of CJRMKm in different intestinal portions of chickens at days 14, 21 and 28 of infection compared to control. Colonization in cecum (**A**), ileum (**B**), jejunum (**C**) and fecal shedding of CJRMKm (**D**). Results are mean ± SD and letters (a–c) are used to mark the significant differences at various time points compared with control and among the treatment groups at *p* < 0.05.

The chickens were orally administered with LC or LC^+*mcra*^ in order to evaluate their roles in competitively reducing the colonization of the marker-containing strain CJRMKm. Even though, the colonization of CJRMkm was decreased notably by both LC and LC^+*mcra*^ compared to the control group, in various parts of chicken gut, chickens that were fed with LC^+*mcra*^ demonstrated a higher reduction in the colonization of CJRMKm than the LC fed groups. Chickens colonized with LC^+*mcra*^ strain showed a notable reduction in CJRMKm colonization in different portions of intestine specifically ileum and jejunum by at least 1 log or more as compared to the chickens which were fed with wild type probiotic (LC) strain at day 14, 21 and 28 (Fig. 2). When comparing the colonization of CJRMKm in the cecum, we observed that chickens pre-administered with LC or LC^+*mcra*^ showed lower CJRMKm colonization in cecum contents (Fig. 2A). Likewise, both probiotics (LC and LC^+*mcra*^) reduced the number of CJRMKm colonization in ileum contents compared to control group (Fig. 2B). The same pattern was found in jejunum contents compared to control group (Fig. 2C). The notable reduction on CJRMkm gastrointestinal colonization was also found in form of a lower number of CJRMKm fecal shedding when fed with LC or LC^+*mcra*^ (Fig. 2D). Notable effectiveness of LC^+*mcra*^ was observed in chickens after 1st week post-challenged with CJRMKm, at which there was an approximate 0.9 log CFU/g reduction in chicken feces compared to control group. In the following 2 weeks, CJRMKm fecal shedding of LC^+*mcra*^ fed chickens continuously reduced by more than 1.0 log CFU/g and 1.10 log CFU/g at day 21 and 28, respectively, compared to the control group (Fig. 2D).

### Reduction on natural colonization of *C. jejuni* and *S. enterica* in probiotic fed chickens

**Figure 3:**
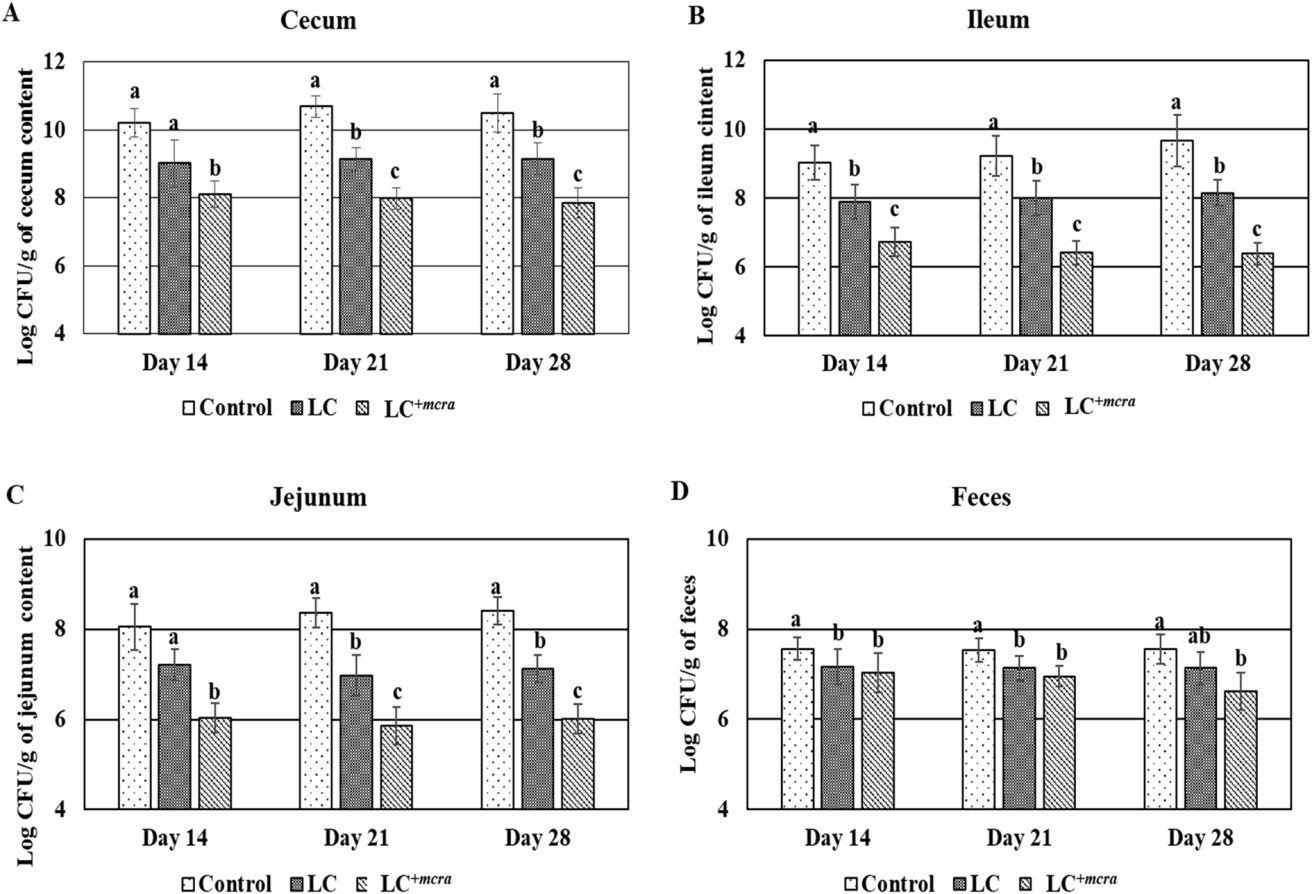
Effect of wild-type probiotic, LC and CLA over producing probiotic, LC^+*mcra*^ on colonization and fecal shedding pattern of *C. jejuni* in different intestinal portions of chickens at days 14, 21 and 28 compared to control. Colonization in cecum (**A**), ileum (**B**), jejunum (**C**) and fecal shedding of *C. jejuni* (**D**). Results are mean ± SD and letters (a–c) are used to mark the significant differences at various time points compared with control and among the treatment groups at *p* < 0.05.

**Figure 4:**
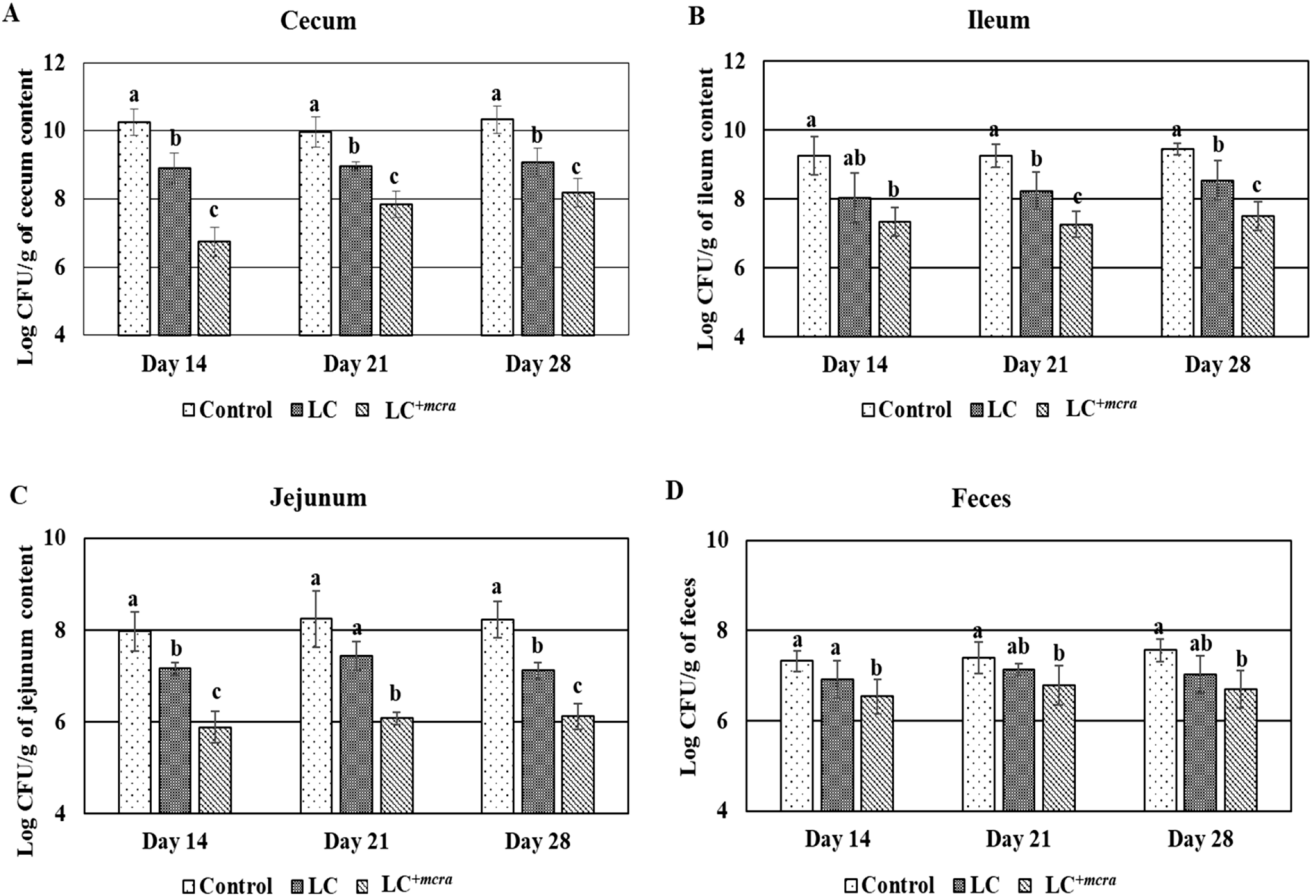
Effect of wild-type probiotic, LC and CLA over producing probiotic, LC^+*mcra*^ on colonization and fecal shedding pattern of *S. enterica* in different intestinal portions of chickens at day 14, 21 and 28 compared to control. Colonization in cecum (**A**), ileum (**B**), jejunum (**C**) and fecal shedding of *S. enterica* (**D**). Results are mean ± SD and letters (a–c) are used to mark the significant differences at various time points compared with control and among the treatment groups at *p* < 0.05.

Chickens pre-treated with either LC or LC^+*mcra*^ showed resistance against the natural colonization of *C. jejuni* and *S. enterica* in cecum, jejunum and ileum, but the chickens pre-treated with LC^+*mcra*^ had a much effective influence on reduction of colonization of both bacterial pathogens (Figs. 3, 4). Specifically, chickens fed with LC^+*mcra*^ showed remarkable reduction in the level of natural colonization of *C. jejuni* in cecum contents, ileum contents and in jejunum contents at days 14, 21 and 28, respectively when compared to the contents of the control groups (Fig. 3A–C). Meanwhile, a consequential decrease in fecal shedding of *C. jejuni* was also detected in LC^+*mcra*^ fed chickens (Fig. 3D). For *S. enterica*, the natural colonization in cecum, ileum and jejunum contents at days 14, 21 and 28, respectively were remarkably lower when pre-treated with LC^+*mcra*^, compared to cecum contents, ileum contents and jejunum contents of the control group (Fig. 4A–C). The LC^+*mcra*^ administration substantially reduced the *S. enterica* fecal shedding as well (Fig. 4D). A similar pattern of reduction was also found in the cecum, ileum, jejunum contents and fecal shedding of *S. enterica* when pre-treated with LC (Fig. 4A–D).

### Reduced level of contamination in water provided to the probiotic fed chickens

**Figure 5:**
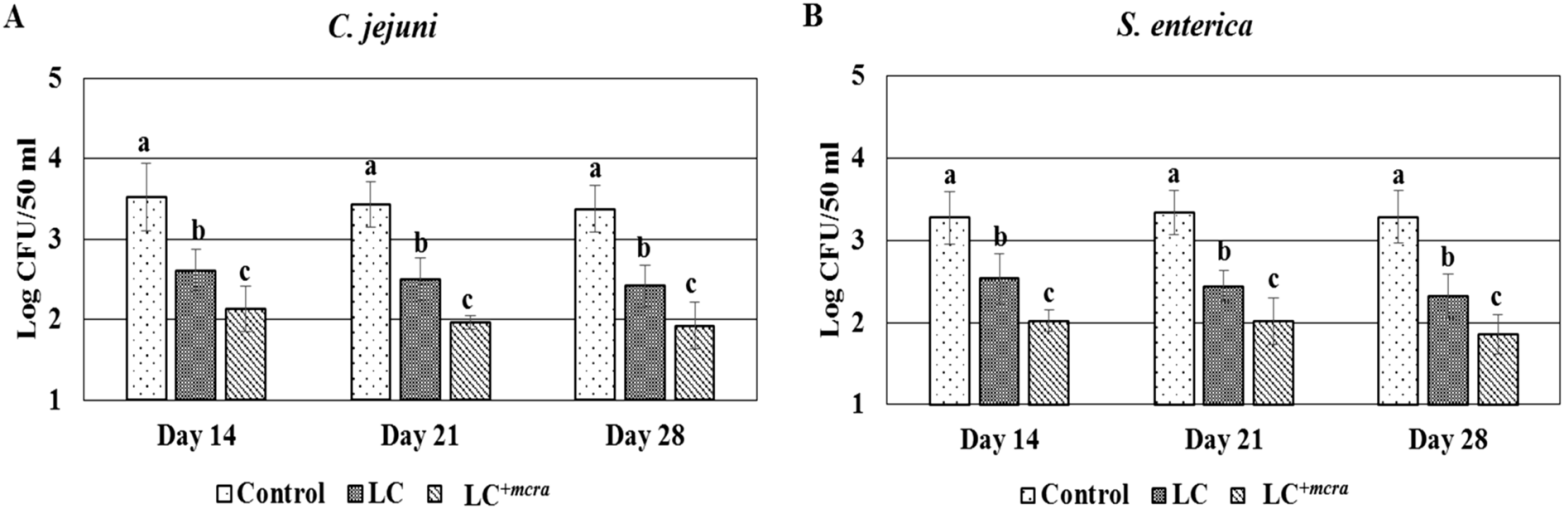
Comparison of water contamination level among the probiotics fed (wild-type probiotic, LC and CLA over-producing probiotic, LC^+*mcra*^) and control groups of chickens. Contamination level of *C. jejuni* (**A**) and *S. enterica* (**B**) in water bottles*.* Results are mean ± SD and letters (a–b) are used to mark the significant differences at various time points compared with control and among the treatment groups at *p* < 0.05.

At different time points (day 14, 21 and 28), water samples were collected to determine the cross-contamination with both *S. enterica* and *C. jejuni*. The contamination level of *C. jejuni* in water was reduced approximately by 1.0 log CFU/50 ml and more than 1.0 log CFU/50 ml in the groups of chickens fed with LC and LC^+*mcra*^, respectively, compared to the control group throughout the experimental period (Fig. 5A). A similar pattern of reduction in *S. enterica* contamination level was also found (Fig. 5B).

### Gut microbiome alteration in the probiotic fed chickens

**Figure 6:**
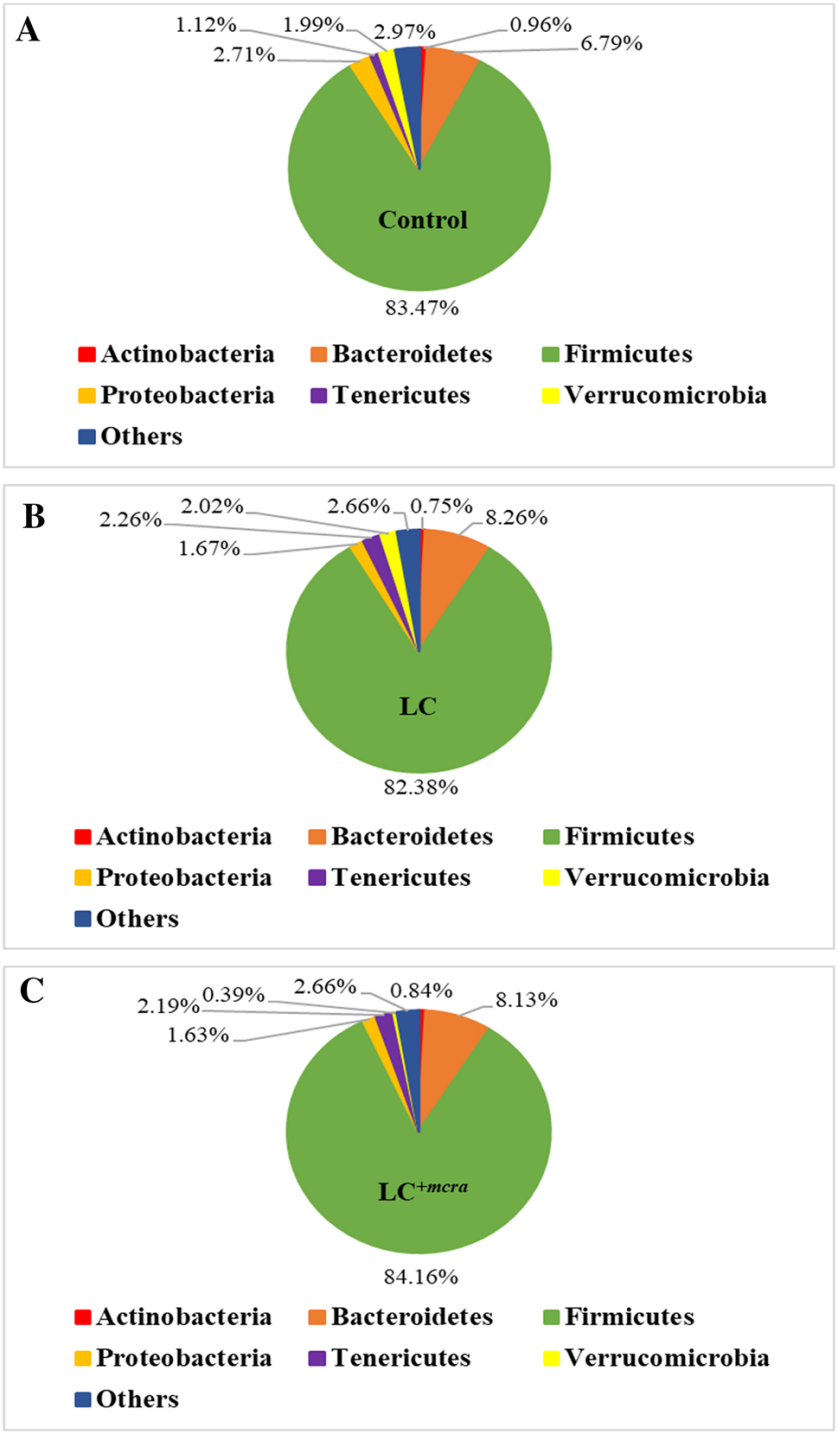
Relative abundance of sequences representing the chicken gut microbiota at phylum level. Comparison of bacterial population of chicken cecal contents collected from different groups at day 28, (**A**) control group, (**B**) wild-type probiotic, LC, fed group and (**C**) CLA over-producing probiotic, LC^+*mcra*^, fed group.

At the phylum level, we observed all three groups of chickens (control, LC fed and LC^+*mcra*^ fed groups) were colonized with abundant number of Firmicutes. Over all 83.47%, 82.38% and 84.16% of Firmicutes were found in control, LC fed group and LC^+*mcra*^ fed group, respectively (Fig. 6). Increased numbers of both Bacteroidetes and Tenericutes was also observed in the LC and LC^+*mcra*^ fed groups compared to the control group. Higher number of Bacteroidetes was also observed in the gut of LC (21.72%) and LC^+*mcra*^ (19.82%) fed chickens compared to the control group. The percentage of Tenericutes was approximately double for both LC and LC^+*mcra*^ fed groups of chickens (Fig. 6). On the other hand, a decrease of the abundance of Proteobacteria in both LC (38.3%) and LC^+*mcra*^ (39.8%) fed groups was observed. In fact, the lowest abundance of Proteobacteria was observed in the LC^+*mcra*^ fed group (Fig. 6). In case of Verrucomicrobia, there was not much difference between control group and LC fed group, but there was a noteworthy reduction of Verrucomicrobia, approximately 80%, in the abundance in LC^+*mcra*^ fed group. For Actinobacteria and other phyla, no remarkable differences were observed among the groups (Fig. 6).

**Figure 7:**
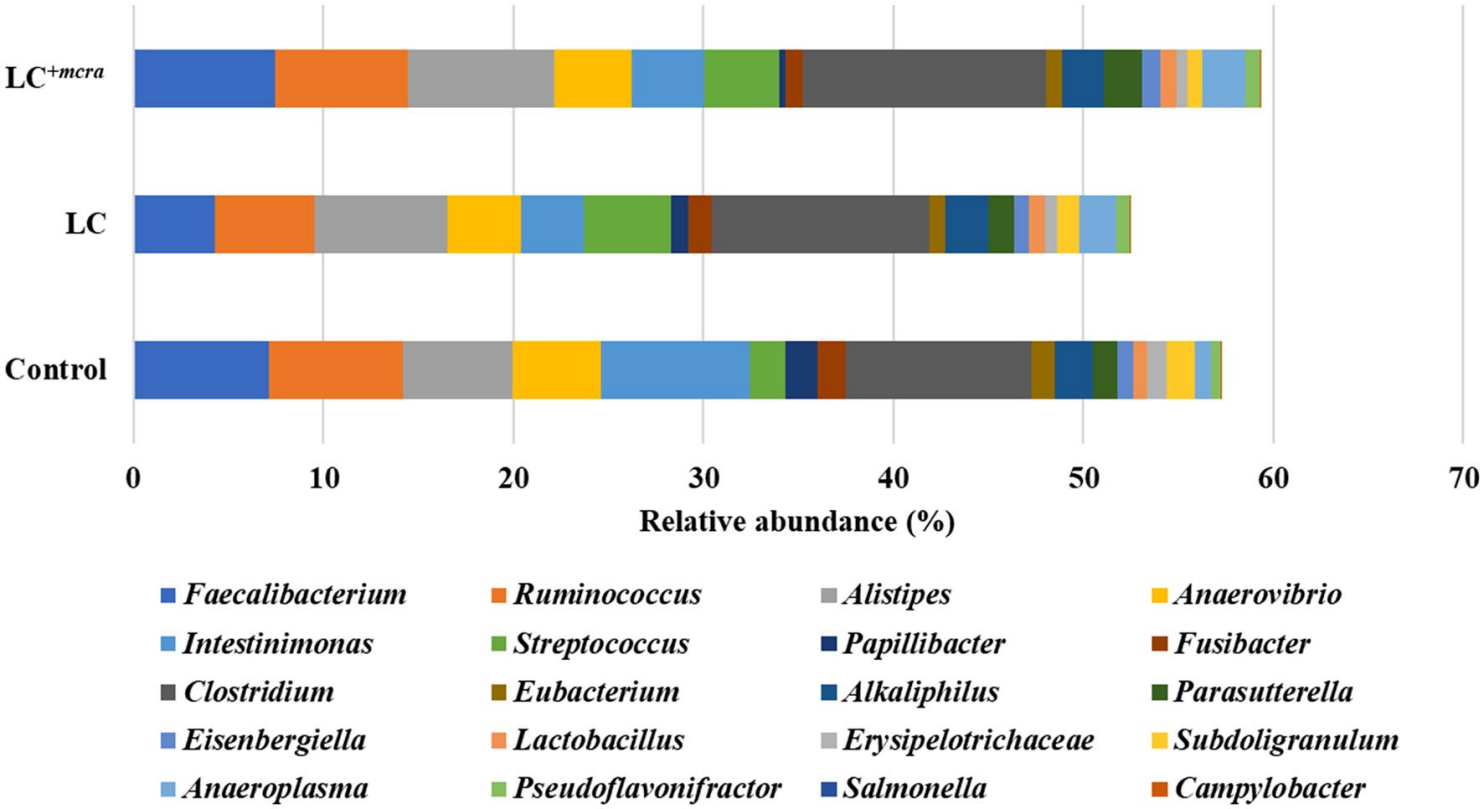
Comparison of bacterial composition at genus level of chicken cecal contents collected at day 28, among control group, wild-type probiotic, LC, fed group and CLA over-producing probiotic, LC^+*mcra*^, fed group.

At the genus level, the distribution of bacteria in the three groups of chickens fed with LC, LC^+*mcra*^ or control showed a distinct variation. For example, there was a decreased abundance in the LC or LC^+*mcra*^ fed groups compared to the control for *Ruminococcus*, *Anaerovibrio*, *Intestinimonas, Papillibacter, Fusibacter, Eubacterium, Erysipelotrichaceae, Subdoligranulum* and most importantly for *Salmonella* and *Campylobacter* (Fig. 7). For *Alistipes, Streptococcus, Clostridium, Alkaliphilus, Parasutterella, Eisenbergiella, Anaeroplasma* and *Pseudoflavonifractor*, there was an increase of the abundance in either the LC or LC^+*mcra*^ fed group compared to the control group (Fig. 7). There was also an increase of *Lactobacillus* in both groups fed with LC and LC^+*mcra*^, as expected. Increase in the genus level abundance of *Anaeroplasma* (2.31- and 2.71-fold increase in groups fed LC and LC^+*mcra*^, respectively) and *Streptococcus* (2.43- and 2.07-fold increase in groups fed LC and LC^+*mcra*^, respectively) was found to be the preeminent difference in groups fed LC and LC^+*mcra*^ when compared to the control. *Pseudoflavonifractor* was also detected with an approximate 1.41 and 1.45-fold increase in LC and LC^+*mcra*^ fed groups, respectively, as compared to the control group. *Lactobacillus* was found to have an increase of around 1.16- and 1.17-fold in groups fed LC and LC^+*mcra*^, respectively, as compared to the control group. On the other hand, the abundance of *Papillibacter* was decreased by 1.86- and 4.85-fold in LC and LC^+*mcra*^ fed groups, respectively; along with a 1.54- and 1.71-fold decrease of *Erysipelotrichaceae* in LC and LC^+*mcra*^ fed groups, respectively, as compared to the control group. Colonization of *Salmonella* was decreased by 2.13- and 3.54-fold in groups fed LC and LC^+*mcra*^, respectively, as compared to the control group. The decrease of *Campylobacter* colonization was approximately 3.16-fold in the LC^+*mcra*^ fed group of chickens compared to the control group (Fig. 7).

**Figure 8:**
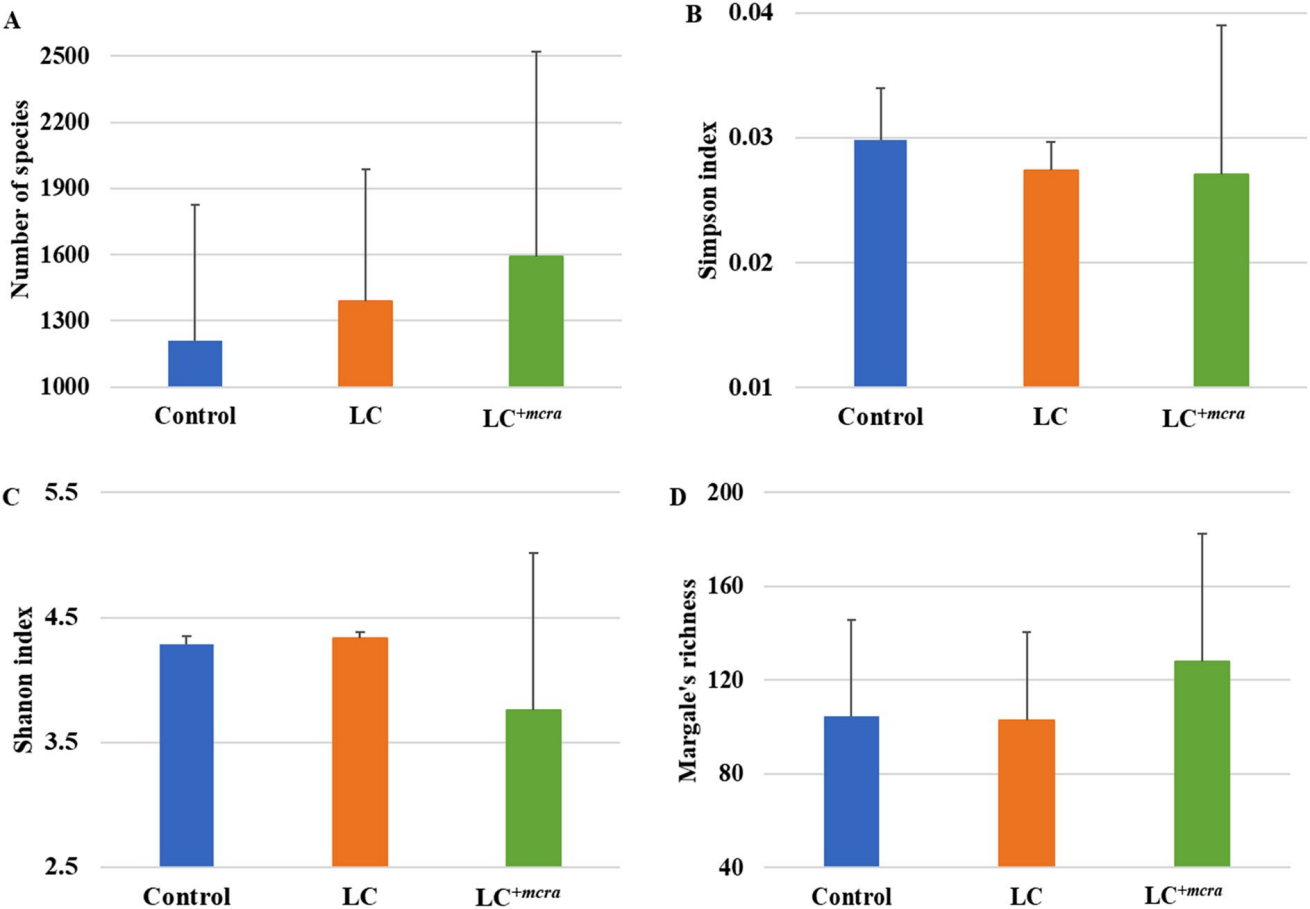
Comparison of chicken gut microbiome at species level. Chicken cecal contents were collected from control, wild-type probiotic, LC, fed group and CLA over-producing probiotic, LC^+*mcra*^, fed groups at day 28. Bacterial diversity at species level (**A**) Number of species, (**B**) Simpson index, (**C**) Shannon index and (**D**) Margalef’s richness was compared. Results are mean ± SD.

We have also compared the diversity of bacterial species among the three groups using alpha indices (Shannon index, Simpson index and Margalef’s richness). There was no significant difference observed in bacterial diversity among the three (control, LC and LC^+*mcra*^ fed) groups. For instance, the number of observed species in both LC and LC^+*mcra*^ fed groups was only numerically higher than the control, with LC^+*mcra*^ group having the highest, an average of 1592 species (Fig. 8A). There was also only numerical difference in Simpson index for control, LC and LC^+*mcra*^ fed group (Fig. 8B). The average of Shannon index of LC^+*mcra*^ fed group was numerically the highest followed by LC fed group and control group (Fig. 8C). The Margalef’s richness was also numerically the highest for LC^+*mcra*^ fed group followed by LC fed group and control group (Fig. 8D).

## Discussion

Previously we showed the antagonistic effect of conjugated linoleic acid over-producing probiotic, LC^+*mcra*^, on enteric bacterial pathogens through in vitro studies^28,30,31^. Here, we verified the effectiveness of CLA over-producing LC^+*mcra*^ against the colonization of the marker-containing CJRMKm strain, as well as on the natural colonization of *C. jejuni* and *S. enterica* in a chicken model. According to our in vitro study, the novel probiotic strain LC^+*mcra*^ demonstrated at least 21 folds more CLA conversion rate in bacterial cell free cultural supernatant and it could also survive longer in comparison with the wild-type LC strain^31^. We also observed a remarkable decrease of *C. jejuni* and *S. enterica* growth in co-culture with LC^+*mcra*^ and in the presence of the cell-free cultural supernatant collected from LC^+*mcra*^. LC^+*mcra*^ could also alter the host cells-*C. jejuni*/*S. enterica* interactions, expression of virulence genes and other virulence properties^30, 31^.

Though, according to literature, efficiency of probiotics in reducing colonization of enteric pathogenic bacteria is yet inconclusive, several groups of research have proposed that the increased production of metabolites from probiotics (e.g., CLA) might improve the overall health benefits of probiotics on host, in addition to the exclusion of enteric pathogens through colonization competition in gut ecosystem^11, 29–31,34,35^. In this study, we used a CJRMKm strain containing kanamycin resistance gene which was inserted into *cdt* of *C. jejuni* chromosome as shown in a previous study demonstrating that deletion of *cdt* did not alter the colonization capability of *C. jejuni* in chicken gut^36^. The 8-day-old chicks challenged with CJRMKm showed a persistent resistance to CJRMKm colonization in their gut through the effect of probiotics. Specifically, probiotic treatments reduced the colonization of CJRMKm from chicken cecum, ileum and jejunum in a time-dependent manner at two, three and four weeks of age. The microbial balance of fecal shedding in the beneficial, commensal and detrimental microbes serves as the crucial indicator of gastrointestinal health^37^, correspondingly we observed the decreased colonization of CJRMKm in both fecal contents and various intestinal contents, as well as an increased colonization trend of probiotic strains. Probiotics themselves were able to limit gastrointestinal bacterial pathogens through competitive exclusion and colonization resistance^9,38–41^.

From the in vivo findings of this study, we observed that both probiotics (LC and LC^+*mcra*^) remarkably diminished *C. jejuni* and *S. enterica* colonization in gastrointestinal portions including cecum, jejunum and ileum, which supported our previous studies^13^. In addition, LC^+*mcra*^ showed more aggressive reductions on the colonization of CJRMKm*, C. jejuni* and *S. enterica* as a result of the effects achieved from its increased amount of CLA conversion^13,31^. Previously, CLA has been reported and associated with antibacterial effect against different enteric pathogens including *C. jejuni* and *S. enterica*, though the distinctive mechanisms of interaction between CLA and bacterial cells are still under investigation^30,31,42^. Furthermore, our in vivo examination based on chicken model also confirmed the protective functions of LC^+*mcra*^ against gastrointestinal bacterial pathogens, supporting the previous in vitro outcomes^29–31^. In mice model, CLA was observed to provide a protective mechanism through both stimulating beneficial bacterial colonization and maintaining the balance in microbial composition in gut ecosystem, which served as the first line defense against foodborne pathogenic bacterial colonization^13^.

The investigation of the chicken normal floral distribution was another goal of this study. Several research groups also reported that CLA-rich diets could stimulate the fatty acids metabolism and preserve homeostatic gut microbiota^43,44^. The balanced gut microbial ecosystem, along with the diversity of microbes, was modified by the introduction of probiotic capable of producing CLA. The researchers also suggested that higher abundances of beneficial bacteria such as *Lactobacillus* and *Bifidobacterium* could influence the gut homeostasis in a positive way that contributes to defending against enteric pathogens^11^. In this study, we found that the most abundant bacterial phyla in both probiotic fed and non-fed group of chickens were Firmicutes and Bacteroidetes. When the chickens were given probiotic either wild-type LC or bioactive LC^+*mcra*^ strain, the abundance of the Firmicutes and Bacteroidetes were increased notably. Previously, Wang et al. also reported a similar finding^45^. When they treated the chickens with probiotics specifically *Lactobacillus*, they also observed that Firmicutes and Bacteroidetes were colonized notably higher in the cecum of the broiler. We also observed the relatively a smaller number of Proteobacteria in both groups of chickens treated with probiotics, specifically in the gut of the LC^+*mcra*^ fed group. Proteobacteria compose with a large number of Gram-negative enteric bacteria including pathogens. By feeding probiotics, several research groups also observed reduced number of Proteobacteria in the animal models^13,46,47^. We also found that *Clostridium, Faecalibacterium, Ruminococcus* and *Alistipes* were the predominant genera in all three groups of chickens which is in agreement with the previous reports of Luo et al. and Shaufi et al.^46,48^. These *Clostridium, Faecalibacterium, Ruminococcus* and *Alistipes* genera are also found to involve in the production of different beneficial metabolites in poultry gut, including SCFA^46^. In our study, we observed that these *Clostridium, Faecalibacterium, Ruminococcus* and *Alistipes* genera were highly abundant in the probiotic fed group chickens specifically in the gut of LC^+*mcra*^ fed chickens.

As two groups of chickens were given orally either LC or LC^+*mcra*^, higher abundance of the *Lactobacillus* species was observed in those groups. Previously Wang et al. also observed that probiotic feeding increased the abundance of the probiotics in poultry^45^. Furthermore, we observed that there was reduced number of Gram-negative bacteria including *Fusibacter, S. enterica* and *C. jejuni* in the gut of probiotic fed chickens. We also found that the decreasing pattern of the Gram-negative bacteria was much more noticeable in the LC^+*mcra*^ fed group than the LC fed group which is agreed with the previous findings of Peng et al.^13^. The abundance and diversity of the available species were also compared in this study. Though there was no statistically significant difference, numerical differences among control, LC and LC^+*mcra*^ fed groups of chickens were observed. Similar finding was also reported by Wang et al.^45^. The diversity of the species largely varies with the heredity, environment and diets, which might be responsible for the nonsignificant difference among the groups of chickens.

In addition to this, reduced numbers of *C. jejuni* and *S. enterica* were observed in the drinking water collected from the water containers in the probiotic-fed groups. This result is supported by our previous studies where we reported LC^+*mcra*^ inhibited *C. jejuni*, *S. enterica* and *E. coli*^13,28–31^. The survival and persisting ability of *C. jejuni* in water has been reported, which means that reducing survivability in drinking water of chicken may limit both the horizontal transfer of this bacterial pathogen to other chicks and the colonization of *C. jejuni* in flocks^49^.

It has been reported that antibiotic growth promoter limits both the prevalence of *C. jejuni* at the pre-harvest level in conventional poultry farms as well as the poultry carcass cross-contamination with *C. jejuni* in conventionally processed chicken meat when compared to organic processors^5,50^. Previously, we observed that berry pomace extracts decreased the natural colonization of *C. jejuni* and colonization of marker-containing CJRMKm strain in chicken cecum remarkably with positive modulation of chicken gut microbiome^51–53^. Similarly, here we also observed noteworthy decrease in CJRMKm, *C. jejuni* and *S. enterica* in different portions of chicken gastrointestinal tract in the probiotics fed chicks (both wild-type and CLA over-producing LC) with the LC^+*mcra*^ having a substantially better performance. In water samples of both probiotics fed groups, there was reduction in the cross-contamination with *C. jejuni* and *S. enterica*. Therefore, the combination of berry pomace extract and LC^+*mcra*^ can be a potential approach for reducing the colonization of *C. jejuni* and *S. enterica* as well as improving the growth of chickens, but further investigation is needed.

Conclusively, this study indicated the effectiveness of CLA over-producing probiotic strain, LC^+*mcra*^, on the colonization of both marker-containing CJRMKm strain and naturally colonized *C. jejuni* and *S. enterica* in various sections of chicken intestine along with the modulation of gut microbiota towards healthier gut. Specifically, chickens fed with LC^+*mcra*^ daily for 1 week showed a notable reduction on the colonization of CJRMKm. Natural colonization of chicken gut and fecal shedding with both *C. jejuni* and *S. enterica* were also observed to be notably reduced by probiotics feeding. Though the study was conducted in controlled laboratory environment, the outstanding roles of LC^+*mcra*^ provides a promising option for reducing the colonization of the most predominant zoonotic pathogens, specifically *S. enterica* and *C. jejuni* in chicken gut, which may eventually lead to lower incidence of cross-contamination in poultry products. Thus, LC^+*mcra*^ may have a beneficial effect on the quality of the poultry products and retain consumer satisfaction with regards to the safety of the poultry products and reducing the risk of zoonotic infections.

## Methods

### Bacterial strains and growth conditions

*Lactobacillus casei* (ATCC 334) (LC) and *Campylobacter jejuni* RM1221 (ATCC BAA-1062) were used in this study. To distinguish from other *C. jejuni*, a marker-containing Kanamycin cassette was inserted into the chromosomal DNA of *C. jejuni* and named as ‘CJRMKm’^52,53^. CJRMKm was grown in Karmali agar (Himedia, India) with selective supplements (Himedia, India) under microaerophilic conditions, in presence of 10% CO_2_, 5% O_2_ and 85% N_2_ at 37 °C. Over-production of conjugated linoleic acid in LC, was achieved by incorporating the myosin cross reactive antigen gene (*mcra*) in LC chromosome giving it the name of ‘LC^+*mcra*^’^13,28–31^. Both LC and LC^+*mcra*^ were grown at 37 °C for 18 h in presence of 5% CO_2_ (Thermo Fisher Scientific Inc., USA) on MRS (de Man Rogosa Sharpe) agar (EMD Chemicals Inc., USA).

### In vivo study design and chicken model

Experiments in the chicken model were carried out up to 4 weeks, in duplicate trials, using 934-1 Isolator Units (Dye Sheet Matter, USA), which were equipped with HEPA filter and air circulation system. Each animal trial consisted with 60 SPF chicks (one day-old), obtained from Charles River, USA. Institutional Animal Care and Use Committee (IACUC, protocol number R-16-33) recommended guidelines were followed for chicken husbandry. Commercially available crumbles without antibiotic supplementation (Purina Animal Nutrition, USA) were used as chicken feed. Chickens were divided into 3 groups (20 chickens per group) which corresponded to their respective 934-1 Isolator Units (Dye Sheet Matter, USA) (Tables 1, 2). For each treated/control group, chickens were further divided into 2 separate sub-groups (10 chickens in each case) and housed in individual 934-1 Isolator Unit (Dye Sheet Matter, USA). Water was given using Gravity-fed water containers.

**Table 1:** Number of chickens allocated in the experimental groups with treatments.

Treatments	Trial 1	Trial 2	Total
Phosphate buffered saline (A)	20	20	40
*L. casei* ATCC334 (B)	20	20	40
Mcra over-expressing *L*. *casei* (C)	20	20	40
Total	60	60	120

**Table 2:** Experimental procedures with timeline (per trial).

Timeline	Number of chickens					
	Daily probiotic colonization	Enteric bacterial challenge	Fecal sample collection	Euthanization	Cecum sample harvest	Live
Day0	–	–	60 (1/chicken)	–	–	60
Day1–Day7	40 (B, C)^a^	–	–	–	–	60
Day8	–	60 (A, B, C)^a^	60 (1/chicken)	–	–	60
Day14	–	–	60 (1/chicken)	18 (6/group)	18 (6/group)	42
Day21	–	–	42 (1/chicken)	18 (6/group)	18 (6/group)	24
Day28	–	–	24 (1/chicken)	24 (8/group)	24 (8/group)	0

^a^A, corresponds to ‘control group’; B. corresponds to the ‘group fed with LC’ and C, corresponds to ‘group fed with LC^+*mcra*^’.

### Feeding probiotics and *C. jejuni* challenge to chicken model

Both the probiotic strains LC and LC^+*mcra*^ were grown at 37 °C overnight in shaking incubator (at 120 rpm) on MRS broth (EMD Chemicals Inc., USA) and suspensions were prepared using PBS. LC and LC^+*mcra*^ solutions (100 μl) containing 10^10^ CFU/ml were fed to chicken groups as shown in Table 2. On day 8, all chickens were challenged with CJRMKm which was grown in Bolton broth (Himedia, India) in presence of 5% fetal bovine serum (FBS) (Corning, USA) with shaking (at 120 rpm) for 30 h under microaerophilic condition (10% CO_2_, 5% O_2_ and 85% N_2_). Suspension of CJRMKm (100 μl) containing 10^9^ CFU/ml was fed to the 8-day-old chicks through oral gavage. Chickens were routinely checked for any physical injury after the gavage administration and then moved back to their respective cages.

### Sample collection from chicken carcass and processing

Euthanization was performed on day 14 and day 21 for 6 chickens per group and on day 28 for the rest of the chickens, with the purpose of evaluating the degree of colonization of probiotics and CJRMKm in the chick cecum, ileum and jejunum. To determine the colonization pattern of probiotics, homogenized cecum, ileum or jejunum contents (~ 200 g) were diluted and then plated on MRS agar (EMD Chemicals Inc., USA) for enumeration. To test the CJRMKm colonization level, cecum, ileum, or jejunum contents (~ 200 g) were homogenized in 1 ml of phosphate buffer saline (PBS) and then plated on Karmali Agar containing selective supplements (Himedia, India) and Kanamycin (100 µg/ml) (Thermo Fisher Scientific, USA) following serial dilution techniques for enumeration. Colonization of *C. jejuni* (challenged and natural) and *S. enterica* (natural) was also determined following standard methods. For enumeration of *C. jejuni* and *S. enterica*, intestinal contents from cecum, ileum or jejunum (~ 200 g) were collected and homogenized in PBS solution (1 ml), diluted and then plated on Karmali Agar with selective supplement and Xylose Lysine Deoxycholate Agar (XLD Agar) (Himedia, India), respectively. Water samples were also collected from the drinking containers of cages. Water samples (50 ml) were collected in centrifuge tubes (VWR, USA) using a sterile 25 ml pipette (VWR, USA). Then the samples were centrifuged (3000 × *g*) for 20 min, bacterial pellets were re-suspended in PBS (1 ml) and plated on Karmali Agar with selective supplement and XLD Agar for enumeration.

### 16S rRNA compositional analysis

For comparison of the chicken gut microbiome, the cecum contents of six 28-day-old chickens from each group were randomly collected for 16S rRNA compositional analysis following the methods previously described by Peng et al.^13^. Briefly, DNA was extracted with QIAamp Fast DNA Stool Kit (QIAGEN, USA). The variable regions (V3 and V4) of 16S rRNA were targeted for phylogenic classifications. DNA libraries were prepared using Nextera DNA Library Preparation Kit and Nextera Index Kit (Illumina, USA) and pooled into equimolar concentration according to the protocol provided by the manufacturers. Sequencing (paired-end: 2 × 300 bp) was preformed using MiSeq v3 600-Cycle Kit (Illumina, USA) by Illumina MiSeq. Sequencing data were processed by MiSeq Reporter-BaseSpace for FASTQ workflow generation (Greengenes database). Demultiplexing was conducted using the perfect index recognition (mismatch = 0) and then by removing PhiX reads. A total 8,509,169 quality-filtered reads were used for analysis of the 16S rRNA composition. The data were analyzed to determine the differences of relative abundance at phylum, genus and species (number of species, Simpson index, Shannon index and Margalef’s richness) levels among three groups (control, LC or LC^+*mcra*^ fed groups) following standard protocol.

### Statistical analysis

For statistical analysis, cecum, ileum or jejunum from a bird was considered as an experimental unit. The data were ranked, and ANOVA was used to differentiate the level of colonization (CFU/g cecum, ileum or jejunum contents). For comparison of the mean ranks, Tukey’s test was used. Differences in the water samples were compared by performing ANOVA and Tukey’s test was used for comparison of mean ranks.

### Ethical approval and informed consent

Institutional Animal Care and Use Committee (IACUC) approval was taken for the chicken trials (Protocol Number R-16-33). IACUC recommended guidelines were followed for chicken husbandry.
